# Retrograde balloon aortic valvuloplasty with the newly invented Inoue balloon for aortic stenosis accompanied by severe heart failure: A case report

**DOI:** 10.1002/ccr3.3928

**Published:** 2021-02-12

**Authors:** Kenichi Ishizu, Tomohiro Kawaguchi, Shinichi Shirai, Kenji Ando

**Affiliations:** ^1^ Department of Cardiovascular Medicine Kokura Memorial Hospital Kitakyushu Japan

**Keywords:** balloon aortic valvuloplasty, aortic stenosis, heart failure

## Abstract

Retrograde balloon aortic valvuloplasty using the newly invented Inoue balloon was one of the feasible and safe therapeutic options in a hemodynamically unstable patient having aortic stenosis with reduced left ventricular ejection fraction complicated with bacterial infection.

## Graphical abstract

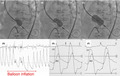

## 1 INTRODUCTION

Aortic stenosis (AS) is a common valvular heart disease in the elderly.^1^ Balloon aortic valvuloplasty (BAV) was introduced as an alternative to aortic valve replacement in 1986; it has played a limited role in the treatment of patients with severe AS owing to complication risks, lack of durability, and its minor impact on long‐term survival.^2^, ^3^ However, in the era of transcatheter aortic valve replacement (TAVR), BAV is frequently utilized to assess the therapeutic response of a reduction in aortic gradient as a bridge procedure to TAVR for selected high‐risk patients who cannot be immediate candidates for TAVR; thus, acute effectiveness and safety are required for BAV.^4^, ^5^ Recently, the Inoue balloon for use in retrograde BAV (Toray, Tokyo, Japan) was newly invented. The hourglass shaped balloon has multiple advantages, including stable fixation and multistage inflation characteristics, and therefore it does not impose any rapid ventricular pacing requirements to fix the balloon position during inflation, compared with cylindrical conventional balloons. We describe the BAV procedure which was safely performed using the newly invented Inoue balloon for retrograde approach in a high‐risk patient.

## 2 CASE

**FIGURE 1:**
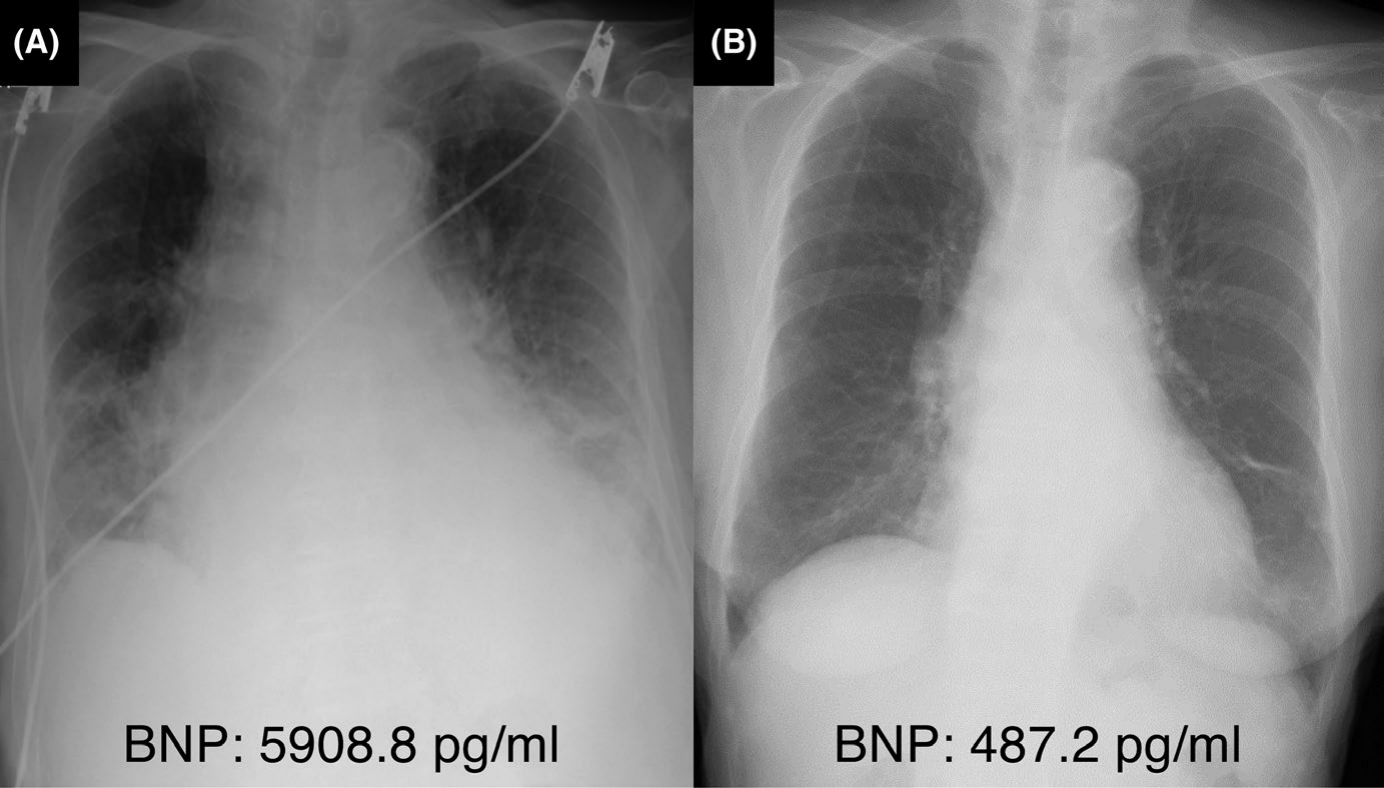
Chest radiographs and the brain natriuretic peptide (BNP) levels before and after retrograde balloon aortic valvuloplasty (BAV). (A) Preprocedural chest radiograph at day 1 showing severe pulmonary congestion with a butterfly shadow. (B) Postprocedural chest radiograph at day 8 showing resolution of pulmonary edema.

**FIGURE 2:**
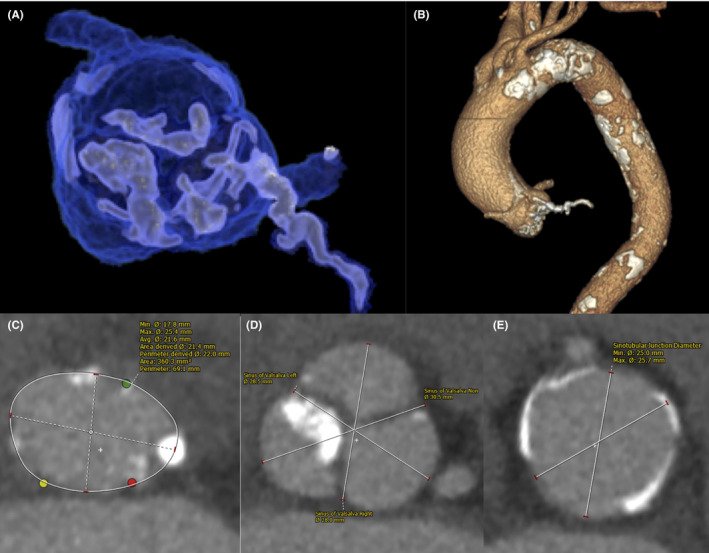
Multidetector computed tomography (MDCT) images of aortic complex. Three‐dimensional reconstruction of MDCT showing a severely calcified aortic valve with left ventricular outflow tract calcification (A) and aortic running (B). Multiplanar reconstruction of MDCT showing an aortic annular area of 360.3 mm^2^ (C), sinus of Valsalva diameter of 28.5 mm at the left coronary cusp, 28.0 mm at the right coronary cusp, 30.5 mm at the noncoronary cusp (D), and sinotubular junction maximum and minimum diameter of 25.7 mm and 25.0 mm (E).

A 95‐year‐old woman with a history of hypertension was referred to our hospital with signs of progressive dyspnea. Upon arrival, she was tachypneic (respiratory rate: 27 breaths/min), hypotensive (blood pressure: 95/67 mm Hg), and tachycardic (heart rate: 110 beats/min). She had high‐grade fever (body temperature: 38.5℃) and her oxygen saturation was 83% on room air. Physical examination revealed coarse crackling and wheezing in both lungs, a Levine IV/VI systolic murmur at the upper right sternal border, and bilateral lower extremity pitting edema. Laboratory test results were normal except for B‐type natriuretic peptide levels of 5908.8 pg/mL, creatinine levels of 1.97 mg/dL, C‐reactive protein levels of 4.2 mg/dL, white blood cell count of 12 000/μL, hemoglobin levels of 115 g/L, albumin levels of 23 g/L, and pyuria with nitrituria. A chest radiograph showed pulmonary congestion with a butterfly shadow and an increased cardiothoracic ratio (Figure 1A). Transthoracic echocardiography revealed severe AS with severely decreased left ventricular ejection fraction (LVEF: 30.3%) and stroke volume (SV index to body surface area: 22 ml/m^2^) (Video S1). The aortic valve area calculated with the continuity equation was 0.38 cm^2^ with a mean transaortic pressure gradient of 45.2 mm Hg. Pulmonary hypertension was suggested by a systolic tricuspid regurgitation pressure gradient of 47.4 mm Hg. Multidetector computed tomography demonstrated a severely calcified aortic valve with aortic annulus area of 360.3 mm^2^ with the maximum diameter of 25.4 mm and the minimum diameter of 17.8 mm (Figure 2).

The patient suffered from severe heart failure owing to low‐flow‐high‐gradient AS with reduced LVEF and urinary tract infection. Noninvasive ventilatory support and intravenous furosemide administration were ineffective and urgent intervention for AS was required before the results of blood cultures were available. Our heart team dismissed the option of a surgical aortic valve replacement because of advanced age and high‐surgical risk (Society of Thoracic Surgeons Predicted Risk of Mortality of 9.217%).^1^ In addition, the patient was considered not to be an immediate candidate for TAVR because of the risks of subsequent endocarditis in the case of prosthesis implantation. Accordingly, we decided to perform a retrograde BAV as a bridge to TAVR.

**FIGURE 3:**
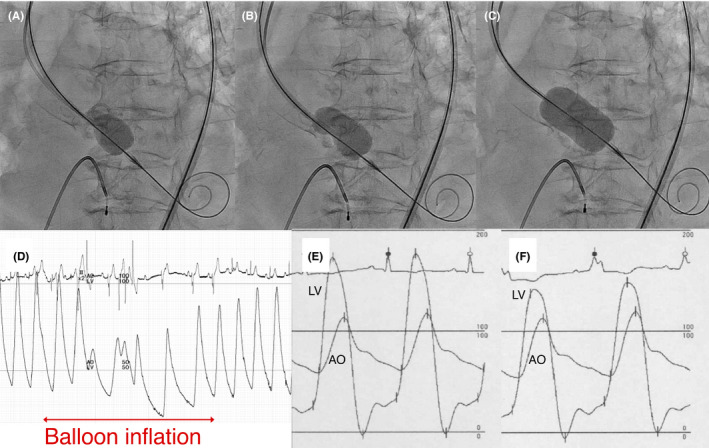
Retrograde balloon aortic valvuloplasty using the Inoue balloon. Fluoroscopic images showing an inflated proximal portion of Inoue balloon (A), stable fixation of the Inoue balloon with its hourglass shape (B), and a fully inflated Inoue balloon (C). (D) Polygraph showing immediate recovery of blood pressure after balloon deflation. (E) Pressure tracing before BAV showing a mean pressure gradient of 51.5 mm Hg. (F) Pressure tracing after BAV showing a mean pressure gradient of 32.2 mm Hg. AO, aorta; LV, left ventricle

Retrograde BAV was performed at day 3. To avoid rapid ventricular pacing considering the severely decreased LVEF, the newly invented Inoue balloon for retrograde BAV use was utilized (Figure 3A‐C, Video S2). Systemic blood pressure decreased only during balloon inflation and recovered several seconds after balloon deflation (Figure 3D). After six times inflations of the 20‐mm Inoue balloon in the absence of rapid ventricular pacing, an adequate acute gain was achieved without any complications (Figure 3E,F). Transthoracic echocardiography performed after the procedure showed improvement in LVEF to 41.1% and the aortic valve area of 0.60 cm^2^ with a mean transaortic pressure gradient of 36.9 mm Hg. The patient's urine output immediately increased after BAV, and her heart failure was completely compensated at day 8 (Figure 1B). After the signs of infection improved, transfemoral‐TAVR with the 23‐mm SAPIEN 3 (Edwards Lifesciences, Irvine, California) was performed at day 23 (Video S3), and the patient was ambulatorily discharged after 4 days.

## 3 DISCUSSION

Earlier reports on the feasibility of the newly invented Inoue balloon for retrograde BAV are scarce. Moriki et al^6^ reported the hemodynamical stability during inflation of the Inoue balloon as predilatation for TAVR; however, there are no available reports regarding retrograde use of the Inoue balloon as a bridge BAV to TAVR. Generally, the Inoue balloon is extensively used for percutaneous transcatheter mitral commissurotomies or antegrade BAVs and has numerous advantages, including stable fixation, multistage inflation, and no requirements for rapid ventricular pacing, compared with conventional balloons. The antegrade BAV using the Inoue balloon reportedly resulted in a greater increase in the postprocedural valve area and a reduction in vascular complications and the risk for stroke, compared with the retrograde BAV using the conventional balloon.^7^ However, the antegrade approach itself is a more technically complicated and demanding procedure because it requires septal puncture and antegrade passage of a wire loop through the circulation. In addition, antegrade approach via femoral vein had lower accessibility to rescue TAVR than retrograde approach via femoral artery. Recently, the new Inoue balloon, which has a longer and thinner shaft, and a more elliptical tip, has invented and utilized for retrograde BAV in Japan.

In our case, we decided to urgently perform the retrograde BAV procedure with the Inoue balloon for three reasons. First, the patient was hemodynamically unstable owing to the severe AS and ongoing bacterial infection despite of the intensive medical therapy. Although there are no guidelines on TAVR for patients with bacterial infection, sepsis during the index TAVR hospitalization was reported to be associated with significantly higher rates of prosthetic valve endocarditis (PVE).^8^ BAV without the need for prosthetic implantation is a reasonable choice in our case in order to avoid subsequent PVE. Second, the patient's severe calcified aortic valve was risky for acute, significant aortic regurgitation post‐BAV which could be resolved by rescue TAVR. Thus, we selected retrograde BAV with higher accessibility to transfemoral‐TAVR than antegrade BAV. Third, the patient's LVEF was significantly reduced. Previous studies reported that a longer ventricular pacing duration was associated with morbidity and mortality, particularly in patients with low LVEF.^9^, ^10^ To avoid rapid ventricular pacing, the Inoue balloon rather than the conventional balloon was utilized in retrograde BAV for our case.

We describe a feasible and safe retrograde BAV case using the newly invented Inoue balloon in a hemodynamically unstable patient having AS with reduced LVEF complicated with bacterial infection.

## CONFLICT OF INTEREST

Shinichi Shirai, MD, is the proctor of transfemoral‐TAVR for Edwards Lifesciences and Medtronic. The other authors have no potential conflict of interest relevant to this article.

## AUTHOR CONTRIBUTIONS

KI: (Data curation: Lead; Writing ‐ original draft: Lead). TK: (Data curation). SS: (Writing ‐ review & editing: Lead). KA: (Supervision: Supporting).

## CONSENT

The authors confirm that written consent for submission and publication of this case including image(s) and associated text has been obtained from the patient in line with COPE guidance.

## Supporting information

Video S1

Video S2

Video S3
